# Liver resection for hepatocellular carcinoma in elderly patients: does age matter?

**DOI:** 10.1186/s12893-024-02528-7

**Published:** 2024-09-05

**Authors:** Ahmed Shehta, Mohamed Medhat, Ahmed Farouk, Ahmed Monier, Rami Said, Tarek Salah, Amgad Fouad, Mahmoud Abdelwahab Ali

**Affiliations:** 1 Gastrointestinal Surgery Center, Department of Surgery, Faculty of Medicine, Mansoura University, Gehan Street, Mansoura, 35516 Egypt; 2 Liver Transplantation Unit, Gastrointestinal Surgery Center, College of Medicine, Mansoura University, Mansoura, Egypt

**Keywords:** Hepatocellular carcinoma, Liver resection, Elderly patients, Survival

## Abstract

**Background:**

Evaluation of the influence of the age of the patients upon the outcomes of liver resection (LR) for hepatocellular carcinoma (HCC).

**Methods:**

HCC patients who underwent LR between 2010 and 2020 were analyzed. They were divided into 3 groups depending on the patient’s age. Group I (patients less than 60 years), Group II (patients between 60 and 69 years), and Group III (patients equal to or more than 70 years).

**Results:**

364 patients were included. A significantly higher serum bilirubin and alpha feto-protein were noted in Group I and serum creatinine was noted in Group III. The study groups did not show any significant differences regarding HCC site, number, macrovascular invasion, the extent of LR, Pringle maneuver, and perioperative blood transfusions. Longer operation time was found in Groups II and III, while more blood loss was noted in Group (I) Group I patients had longer hospital stays. Higher postoperative morbidities were noted in both Group I and Group (II) Higher incidence of post-hepatectomy liver dysfunction was noted in Group I. More early mortalities were found in Group I, related to liver failure. We did not experience early mortality in Group (III) Late Mortalities occurred in 117 patients (32.1%). HCC recurrence occurred in 165 patients (45.3%). Regarding the overall- and tumor-free survival, we did not experience any significant differences among the 3 groups (Log Rank: *p* = 0.371 and 0.464 respectively).

**Conclusions:**

Curative LR can be safely performed in selected elderly patients with HCC. An advanced patient’s age should not be considered as a contraindication for curative LR.

## Background

With improvement of the average life expectancy during the recent decades, management of the elderly with various types of neoplasms is a rising global concern. Recent studies had shown the rising number of elderly patients admitted to the surgical wards with more than 60% of them are 65 years old or older [1, 2]. It is well known that old age is a well-established risk factor for the development of hepatobiliary malignancies including hepatocellular carcinoma (HCC). Globally, the number of elderly HCC patients has dramatically increased. In developed countries, like Japan, the average age of HCC diagnosis has increased to 66.4 years among men and 69.9 years among women. Also, liver resection is frequently performed for elderly patients with more than 50% of them older than 70 years [3, 4].

HCC is considered as one of the most lethal abdominal tumors. HCC is the second most common cause of tumor-associated death worldwide [5, 6]. For HCC patients with adequate hepatic parenchymal reserve, liver resection remains the most appropriate treatment modality [7, 8]. Several potential risk factors were associated with the prognosis of HCC patients undergoing liver resection including size, multifocality, macrovascular invasion, serum tumor markers and perioperative transfusions [9]. In the same context, age has been conveyed to have an essential role in the prognosis of HCC patients. It is hard to determine whether liver resection for HCC is beneficial for elderly patients because of the associated comorbidities which may result in higher incidence of postoperative morbidities and mortality. However, the perioperative and long-term outcomes were heterogenous between different studies dealing with management of older patients with HCC. Some studies have shown comparable outcomes of younger and older HCC patients after curative liver resection [10–12]. On the other hand, other studies had shown significantly worse overall survival among older HCC patients following curative liver resection [13].

Age groups vary between the different countries, which reflect the differences in the social conditions and the functional ability related to the workforce. Generally, the definition of elderly people is usually linked to the age of retirement which is associated with significant reduction of the physical activity and psychological condition [14]. The Egyptian government applies the age of 60 years for defining the elderly population, which represents the age of retirement in the Egyptian Government [15, 16].

Data about the perioperative and survival outcomes of elderly patients undergoing liver resection from Egypt are limited.

The purpose of the current study is to assess a single center experience of liver resection for HCC in elderly patients (exceeding 60 years) in comparison with younger patients. In addition, to assess how the age of the patients influences the perioperative and survival results following liver resection for HCC.

## Methods

### Study design

A retrospective review was conducted on the data of HCC patients who underwent liver resection at the Gastro-intestinal Surgery Center, Mansoura University, Egypt, between January 2010 and December 2020. Depending on their age at the time of operation, the patients were split into three groups. Patients in Group I was under 60 years old, patients in Group II were between 60 and 69 years old, and patients in Group III were equal to or older than 70 years old.

Before the procedure, the HCC patient gave us their informed consent. We obtained the clearance of the Faculty of Medicine Institutional Review Board and Ethical Committee, Mansoura University (Code R.22.12.2000).

### Preoperative evaluation

Preoperative workup was described elsewhere [17–19]. HCC patients were considered good candidates for liver resection if adequate liver reserve is available, absence of significant portal hypertension, absence of distant metastases, with good performance status (American Society of Anesthesiologists score less than III) [20].

### Surgical procedure

The method of liver resection surgery has been previously documented [18, 19]. The Brisbane 2000 classification was used to determine the extent of liver resection [21]. Major liver resections were carried out for HCC patients with big tumors or tumors near major hepatic blood vessels, assuming there was enough future remnant liver [22].

For some selected HCC patients who required major hepatectomy with borderline liver functions, liver volumetry was performed. Otherwise, localized hepatectomy was preferred. For parenchymatous transection, a combination of Kelly-clamp method and ultrasonic device was routinely utilized. Pringle’s maneuver was utilized in selected non-anatomical liver resections. Intraoperative ultrasonography and intraoperative cholangiography were utilized when required.

### Postoperative care and follow up

The postoperative care and follow up protocol had been described before elsewhere [17–19]. Patients underwent standard laboratory (including blood picture, liver functions, kidney function and serum electrolytes) and radiographic evaluations (abdominal ultrasound) while being followed in the intensive care unit or transferred to the general ward. Patients were encouraged for early ambulation and resumption of oral intake.

After their discharge, patients were routinely checked on in the outpatient clinic. Patients received routine laboratory testing, including serum alpha fetoprotein, abdominal radiography, including abdominal ultrasound and triphasic abdominal computed tomography, as well as routine history and clinical examination.

### Definitions

The Clavien-Dindo classification was used to prospectively record and grade postoperative morbidities [23]. Hemorrhage, biliary leakage, and liver dysfunction were defined using the criteria developed by the International Study Group on Liver Surgery [24–26]. Early mortality was defined as postoperative mortality that occurred during the first ninety days following surgery. From the time of surgery to the time of a tumor recurrence or the last follow-up appointment, the disease-free survival (DFS) was calculated. The estimation of overall survival (OS) considered the time interval between the surgery and the patient’s death or the last follow-up appointment.

### Statistical analysis

The median and range were used to describe continuous data. Numbers and percentages were utilized to handle categorical data. The three study groups were compared using the chi-square and ANOVA tests. To compare the two research groups, pairwise comparisons were used.

The OS and DFS rates were evaluated using the Kaplan-Meier technique. The study’s groups were compared using the Log-Rank test. The software program of the Statistical Package of Social Sciences (SPSS – version 22, IBM, Chicago, IL, USA) was utilized for doing statistical analysis. A statistically significant result was defined as a p value of less than 0.05.

## Results

### Demographic data

During the study period, 364 HCC patients underwent liver resection and were enrolled in the current study. There were 157 patients (43.1%) in Group I, 168 patients (46.2%) in Group II, and 39 patients (10.7%) in Group III.

In Table (1), the demographic data was assembled. Group I had considerably greater preoperative serum bilirubin and alpha fetoprotein levels. Group III had a noticeably higher pretreatment serum creatinine level.

**Table 1:** Preoperative characteristics (INR, international normalized ratio)

	All patients (*N* = 364)	Group I (*N* = 157)	Group II (*N* = 168)	Group III (*N* = 39)	*P*
Age (years)	61 (18–80)	55 (18–59)	64 (60–69)	72 (70–80)	0.001
Gender					0.307
Male	292 (80.2%)	121 (77.1%)	137 (81.5%)	34 (87.2%)	
Female	72 (19.8%)	36 (22.9%)	31 (18.5%)	5 (12.8%)	
Complaint					0.343
Accidental	201 (55.2%)	79 (50.3%)	101 (60.1%)	21 (53.8%)	
Pain	159 (43.7%)	77 (49%)	65 (38.7%)	17 (43.6%)	
Mass	4 (1.1%)	1 (0.6%)	2 (1.2%)	1 (2.6%)	
Serum albumin (g/dL)	3.9 (2.2–5.3)	4 (2.4–5)	3.9 (2.2–5.3)	3.8 (2.2–4.7)	0.209
Serum bilirubin (mg/dL)	0.7 (0.3–11.2)	0.8 (0.3–2)	0.6 (0.4–11.2)	0.6 (0.4–1.8)	0.001
Serum alanine amino-transferase (IU/L)	39 (20–512)	40 (20–280)	36.5 (20–512)	38 (20–114)	0.474
Serum aspartate amino-transferase (IU/L)	47 (20–330)	52 (20–240)	45.5 (20–330)	39 (20–180)	0.055
Serum INR	1 (1–2)	1 (1–1.8)	1 (1–2)	1 (1–1.4)	0.892
Serum platelets count (×10^3^/mL)	150 (34–433)	146 (34–433)	153 (41–349)	163 (58–333)	0.205
Serum creatinine (mg/dL)	0.8 (0.5–3)	0.8 (0.5–1.4)	0.8 (0.5–2.7)	0.9 (0.5–3)	0.015
Serum marker α feto-protein (ng/ml)	31.2 (1–3000)	51 (1.6–2000)	28.5 (1–3000)	15.9 (1–2000)	0.036
Child-Turcotte-Pugh					0.946
grade	356 (97.8%)	154 (98.1%)	164 (97.6%)	38 (97.4%)	
A	8 (2.2%)	3 (1.9%)	4 (2.4%)	1 (2.6%)	
B					
Score of models for end stage liver disease **(45)**	7 (6–17)	7 (6–16)	7 (6–17)	7 (6–13)	0.752
Preoperative serum viral hepatitis C	339 (93.1%)	142 (90.4%)	160 (95.2%)	37 (94.9%)	0.21

Bilirubin: Group I vs. II: *p* = 0.005, Group I vs. III: *p* = 0.003, Group II vs. III: *p* = 1

Creatinine: Group I vs. II: *p* = 0.207, Group I vs. III: *p* = 0.017, Group II vs. III: *p* = 0.329

α feto-protein: Group I vs. II: *p* = 0.352, Group I vs. III: *p* = 0.022, Group II vs. III: *p* = 0.677

### Preoperative data

Radiological and endoscopic data were summarized in Table (2).

**Table 2:** Preoperative investigations (radiology and endoscopy)

	All patients (*N* = 364)	Group I (*N* = 157)	Group II (*N* = 168)	Group III (*N* = 39)	*P*
Liver condition					0.014
Hepatic cirrhosis	347 (95.3%)	144 (91.7%)	164 (97.6%)	39 (100%)	
Normal	17 (4.7%)	13 (8.3%)	4 (2.4%)	0	
Tumor number					0.964
Single	333 (91.5%)	143 (91.1%)	154 (91.7%)	36 (92.3%)	
Multiple	31 (8.5%)	14 (8.9%)	14 (8.3%)	3 (7.7%)	
Tumor location					0.732
Right lobe	38 (10.4%)	20 (12.7%)	15 (8.9%)	3 (7.7%)	
Left lobe	18 (4.9%)	4 (2.5%)	12 (7.1%)	2 (5.1%)	
Left lateral portion	44 (12.1%)	15 (9.6%)	24 (14.3%)	5 (12.8%)	
Liver segment 4	20 (5.5%)	5 (3.2%)	13 (7.7%)	2 (5.1%)	
Right anterior portion	3 (0.8%)	2 (1.3%)	1 (0.6%)	0	
Right posterior portion	18 (4.9%)	9 (5.7%)	8 (4.8%)	1 (2.6%)	
Central location	5 (1.4%)	1 (0.6%)	3 (1.8%)	1 (2.6%)	
Segment 1	6 (1.6%)	2 (1.3%)	3 (1.8%)	1 (2.6%)	
Segment 2	24 (6.6%)	12 (7.6%)	10 (6%)	2 (5.1%)	
Segment 3	44 (12.1%)	21 (13.4%)	18 (10.7%)	5 (12.8%)	
Segment 5	15 (4.1%)	7 (4.5%)	5 (3%)	3 (7.7%)	
Segment 6	63 (17.3%)	23 (14.6%)	32 (19%)	8 (20.5%)	
Segment 7	29 (8%)	14 (8.9%)	11 (6.5%)	4 (10.3%)	
Segment 8	16 (4.4%)	11 (7%)	5 (3%)	0	
Multiple locations	21 (5.8%)	11 (7%)	8 (4.8%)	2 (5.1%)	
Macroscopic portal vein invasion	45 (12.4%)	21 (13.4%)	23 (13.7%)	1 (2.6%)	0.144
Porta hepatis lymph nodes	44 (12.1%)	26 (16.6%)	18 (10.7%)	0	0.013
Upper GIT endoscopy	355 (97.5%)	154 (98.1%)	163 (97%)	38 (97.4%)	0.826
Endoscopy findings					0.438
Esophageal varices	57 (15.7%)	30 (19.1%)	23 (13.7%)	4 (10.3%)	
Compression on the stomach	1 (0.3%)	0	1 (0.6%)	0	

Liver condition: Group I vs. II: *p* = 0.023, Group I vs. III: *p* = 0.075, Group II vs. III: *p* = 1

Porta hepatis lymphadenopathy: Group I vs. II: *p* = 0.124, Group I vs. III: *p* = 0.003, Group II vs. III: *p* = 0.028

### Operative outcomes

Table (3) provided a summary of the operative data. Group I had a higher reported blood loss, but Groups II and III had significantly longer surgery durations.

**Table 3:** Operative outcomes

	All patients (*N* = 364)	Group I (*N* = 157)	Group II (*N* = 168)	Group III (*N* = 39)	*P*
Liver status					0.53
Cirrhosis	344 (94.5%)	146 (93%)	161 (95.8%)	37 (94.9%)	
Normal	20 (5.5%)	11 (7%)	7 (4.2%)	2 (5.1%)	
Tumor location					0.086
Right lobe	187 (51.4%)	91 (58%)	77 (45.8%)	19 (48.7%)	
Left lobe	161 (44.2%)	62 (39.5%)	84 (50%)	15 (38.5%)	
Segment 1	6 (1.6%)	2 (1.3%)	3 (1.8%)	1 (2.6%)	
Both lobes	10 (2.7%)	2 (1.3%)	4 (2.4%)	4 (10.3%)	
Tumor number					0.693
Single	337 (92.6%)	147 (93.6%)	155 (92.3%)	35 (89.7%)	
Multiple	27 (7.4%)	10 (6.4%)	13 (7.7%)	4 (10.3%)	
Vascular invasion	45 (12.4%)	23 (14.6%)	21 (12.5%)	1 (2.6%)	0.121
Biliary invasion	2 (0.5%)	1 (0.6%)	1 (0.6%)	0	0.885
Nearby organ invasion	20 (5.5%)	7 (4.5%)	10 (6%)	3 (7.7%)	0.689
Lymph nodes	25 (6.9%)	12 (7.6%)	13 (7.7%)	0	0.2
Extent of hepatectomy					0.108
Minor	278 (76.4%)	133 (72%)	131 (78%)	34 (87.2%)	
Major	86 (23.6%)	44 (28%)	37 (22%)	5 (12.8%)	
Type of hepatectomy					0.512
Wedge resection	169 (46.4%)	75 (47.8%)	75 (44.6%)	19 (48.7%)	
Segment resection	8 (2.2%)	5 (3.2%)	2 (1.2%)	1 (2.6%)	
Left lateral resection	83 (22.8%)	28 (17.8%)	44 (26.2%)	11 (28.2%)	
Right anterior resection	1 (0.3%)	0	1 (0.6%)	0	
Right posterior resection	4 (1.1%)	1 (0.6%)	3 (1.8%)	0	
Left lobectomy	17 (4.7%)	6 (3.8%)	9 (5.4%)	2 (5.1%)	
Extended left lobectomy	1 (0.3%)	0	1 (0.6%)	0	
Right lobectomy	60 (16.5%)	33 (21%)	25 (14.9%)	2 (5.1%)	
Extended right lobectomy	7 (1.9%)	5 (3.2%)	1 (0.6%)	1 (2.6%)	
Central liver resection	1 (0.3%)	0	1 (0.6%)	0	
Segment 1 resection	6 (1.6%)	2 (1.3%)	3 (1.8%)	1 (2.6%)	
Multiple resections	7 (1.9%)	2 (1.3%)	3 (1.8%)	2 (5.1%)	
Associated portal thrombectomy	6 (1.6%)	3 (1.9%)	3 (1.8%)	0	0.691
Associated extrahepatic biliary resection	2 (0.5%)	1 (0.6%)	1 (0.6%)	0	0.885
Pringle procedure use	62 (17%)	28 (17.8%)	25 (14.9%)	9 (23.1%)	0.443
Total operative duration (hours)	3 (1.2–7)	3 (1.2–7)	3 (1–6)	2 (1.3–4.5)	0.005
Total operative losses (ml)	550 (50–6000)	700 (50–6000)	500 (50–4000)	500 (50–5000)	0.006
Blood transfusion requirement	176 (48.4%)	86 (54.8%)	76 (45.2%)	14 (35.9%)	0.059

Operation duration (hours): Group I vs. II: *p* = 0.195, Group I vs. III: *p* = 0.006, Group II vs. III: *p* = 0.025

Operative losses (ml): Group I vs. II: *p* = 0.029, Group I vs. III: *p* = 0.021, Group II vs. III: *p* = 0.961

### Postoperative outcomes

Table (4) provided a summary of the postoperative data. Group I had longer hospital stay than the other groups. Groups I and II showed a higher incidence of postoperative morbidities. Group I had a higher incidence of liver failure-related early postoperative mortality. It should be mentioned that Group III did not have any early postoperative mortality.

**Table 4:** Postoperative outcomes

	All patients (*N* = 364)	Group I (*N* = 157)	Group II (*N* = 168)	Group III (*N* = 39)	*P*
Intensive care duration (days)	1 (1–22)	1 (1–22)	1 (1–7)	1 (1–3)	0.928
Total hospitalization duration (days)	5 (2–66)	6 (2–66)	5 (3–30)	4 (2–7)	0.007
Morbidity	168 (46.2%)	88 (56.1%)	71 (42.3%)	9 (23.1%)	0.001
Clavien-Dindo					0.768
complications grade					
1	69 (19%)	33 (21%)	31 (18.5%)	5 (12.8%)	
2	48 (13.2%)	26 (16.6%)	20 (11.9%)	2 (5.1%)	
3	30 (8.2%)	14 (8.9%)	14 (8.4%)	2 (5.2%)	
4	2 (0.5%)	2 (1.3%)	0	0	
5	19 (5.2%)	13 (8.3%)	6 (3.6%)	0	
Liver dysfunction	144 (39.6%)	76 (48.4%)	61 (36.3%)	7 (17.9%)	0.001
Liver dysfunction grade					0.071
A	83 (22.8%)	37 (23.6%)	39 (23.2%)	7 (17.9%)	
B	41 (11.3%)	26 (16.6%)	15 (8.9%)	0	
C	20 (5.5%)	13 (8.3%)	7 (4.2%)	0	
Bile leakage	19 (5.2%)	9 (5.7%)	10 (6%)	0	0.299
Collection	18 (4.9%)	8 (5.1%)	7 (4.2%)	3 (7.7%)	0.654
Abdominal hemorrhage	7 (1.9%)	3 (1.9%)	3 (1.8%)	1 (2.6%)	0.95
Surgical site infection	9 (2.5%)	4 (2.5%)	4 (2.4%)	1 (2.6%)	0.995
Intrahepatic Abscess	3 (0.8%)	2 (1.3%)	1 (0.6%)	0	0.664
Vascular complications	5 (1.4%)	3 (1.9%)	2 (1.2%)	0	0.632
Respiratory complications	22 (6%)	11 (7%)	10 (6%)	1 (2.6%)	0.58
Cardiac dysrhythmia	1 (0.3%)	0	1 (0.6%)	0	0.557
Renal complications	4 (1.1%)	2 (1.3%)	2 (1.2%)	0	0.782
Cerebral stroke	1 (0.3%)	1 (0.6%)	0	0	0.516
Ileus	1 (0.3%)	0	1 (0.6%)	0	0.557
Bleeding esophageal varices	1 (0.3%)	1 (0.6%)	0	0	0.516
Operative mortality	19 (5.2%)	13 (8.3%)	6 (3.6%)	0	0.049
Causes of mortalities Liver failure Pulmonary embolism	18 (4.9%) 1 (0.3%)	12 (7.7%) 1 (0.6%)	6 (3.6%) 0	0 0	0.469

Hospital duration (days): Group I vs. II: *p* = 0.17, Group I vs. III: *p* = 0.009, Group II vs. III: *p* = 0.192

Morbidity: Group I vs. II: *p* = 0.013, Group I vs. III: *p* = 0.001, Group II vs. III: *p* = 0.029

Liver dysfunction: Group I vs. II: *p* = 0.027, Group I vs. III: *p* = 0.001, Group II vs. III: *p* = 0.036

Operative mortality: Group I vs. II: *p* = 0.113, Group I vs. III: *p* = 0.047, Group II vs. III: *p* = 0.352

### Pathological outcomes

Pathological data were summarized in Table (5). Perineural invasion was more frequently seen in Group I. Higher incidence of grades I and II was noticed in Group III.

**Table 5:** Pathological outcomes

	All patients (*N* = 364)	Group I (*N* = 157)	Group II (*N* = 168)	Group III (*N* = 39)	*P*
Tumor size (cm)	6 (1.5–20)	6 (1.5–20)	6 (1.5–20)	6.5 (2–15)	0.417
Tumor number					0.445
Single	318 (87.4%)	141 (89.8%)	143 (85.1%)	34 (87.2%)	
Multiple	46 (12.6%)	16 (10.2%)	25 (14.9%)	5 (12.8%)	
Resection margin					0.087
R0	319 (87.6%)	133 (84.7%)	154 (91.7%)	32 (82.1%)	
R1	45 (12.4%)	24 (15.3%)	14 (8.3%)	7 (17.9%)	
Capsular invasion	131 (36%)	52 (33.1%)	67 (39.9%)	12 (30.8%)	0.345
Microvascular invasion	170 (46.7%)	75 (47.8%)	80 (47.6%)	15 (38.5%)	0.551
Perineural invasion	125 (34.3%)	60 (38.2%)	59 (35.1%)	6 (15.4%)	0.026
Tumor Grade					0.029
1	65 (17.9%)	32 (20.4%)	28 (16.7%)	5 (12.8%)	
2	220 (60.4%)	81 (51.6%)	107 (63.7%)	32 (82.1%)	
3	69 (19%)	40 (25.5%)	27 (16.1%)	2 (5.1%)	
4	9 (2.5%)	4 (2.5%)	5 (3%)	0	
No viable tumor	1 (0.3%)	0	1 (0.6%)	0	
Liver background					0.624
Cirrhosis	345 (94.8%)	148 (94.3%)	161 (95.8%)	36 (92.3%)	
Normal	19 (5.2%)	9 (5.7%)	7 (4.2%)	3 (7.7%)	

Perineural invasion: Group I vs. II: *p* = 0.562, Group I vs. III: *p* = 0.008, Group II vs. III: *p* = 0.021

Tumor grade: Group I vs. II: *p* = 0.129, Group I vs. III: *p* = 0.005, Group II vs. III: *p* = 0.201

### Survival outcomes

#### Overall survival

**Fig. 1:**
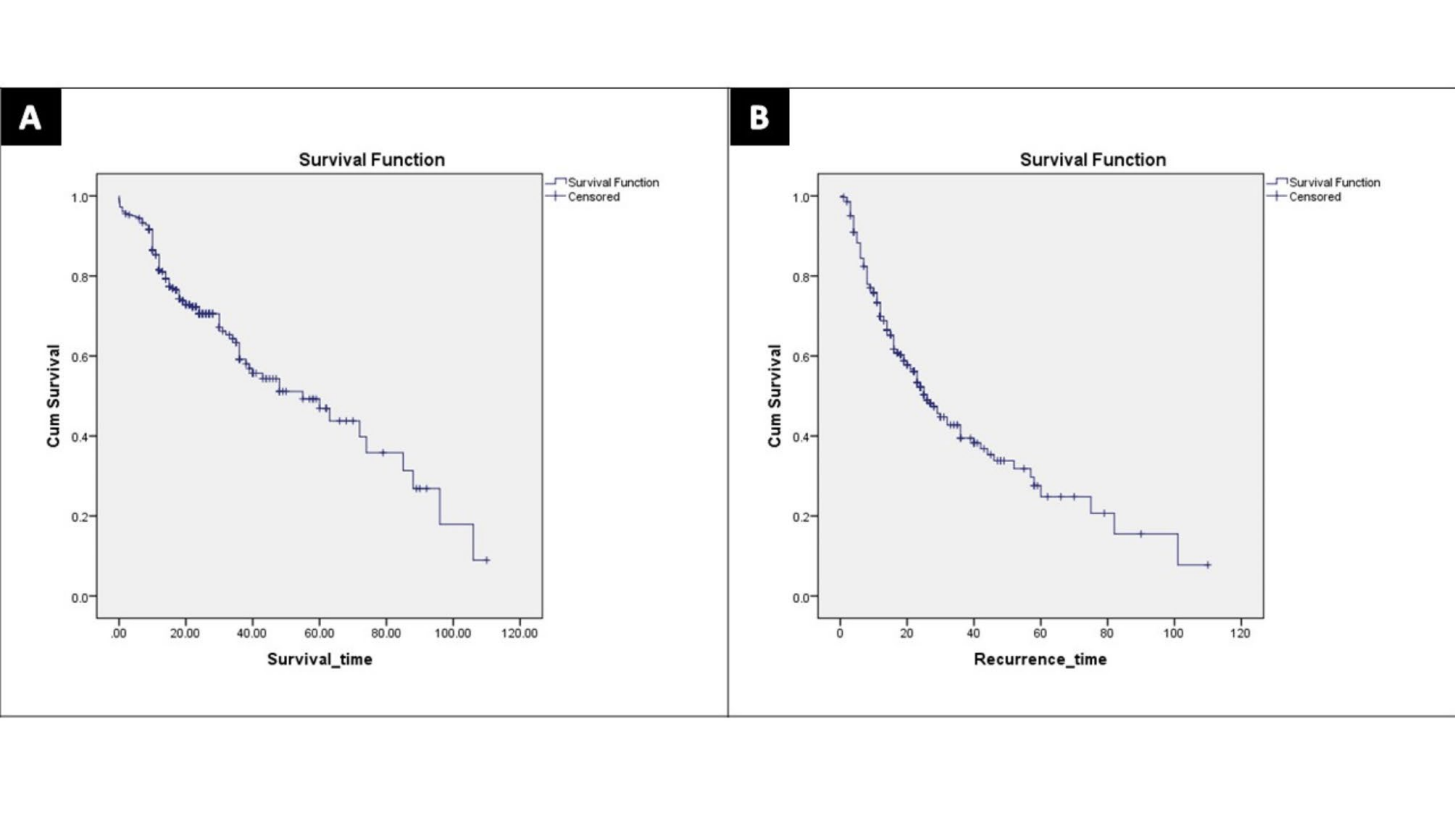
**A**- Overall survival curve of all cases. **B**- Disease-free survival curve of all cases

The median follow-up time was 17 months (4–110 months). Postoperative mortality happened in 117 patients (32.1%). The 1-, 3-, and 5-years OS rates of the whole patients were 85.3%, 64.3%, and 46.9%, respectively (Fig. 1-A).

**Fig. 2:**
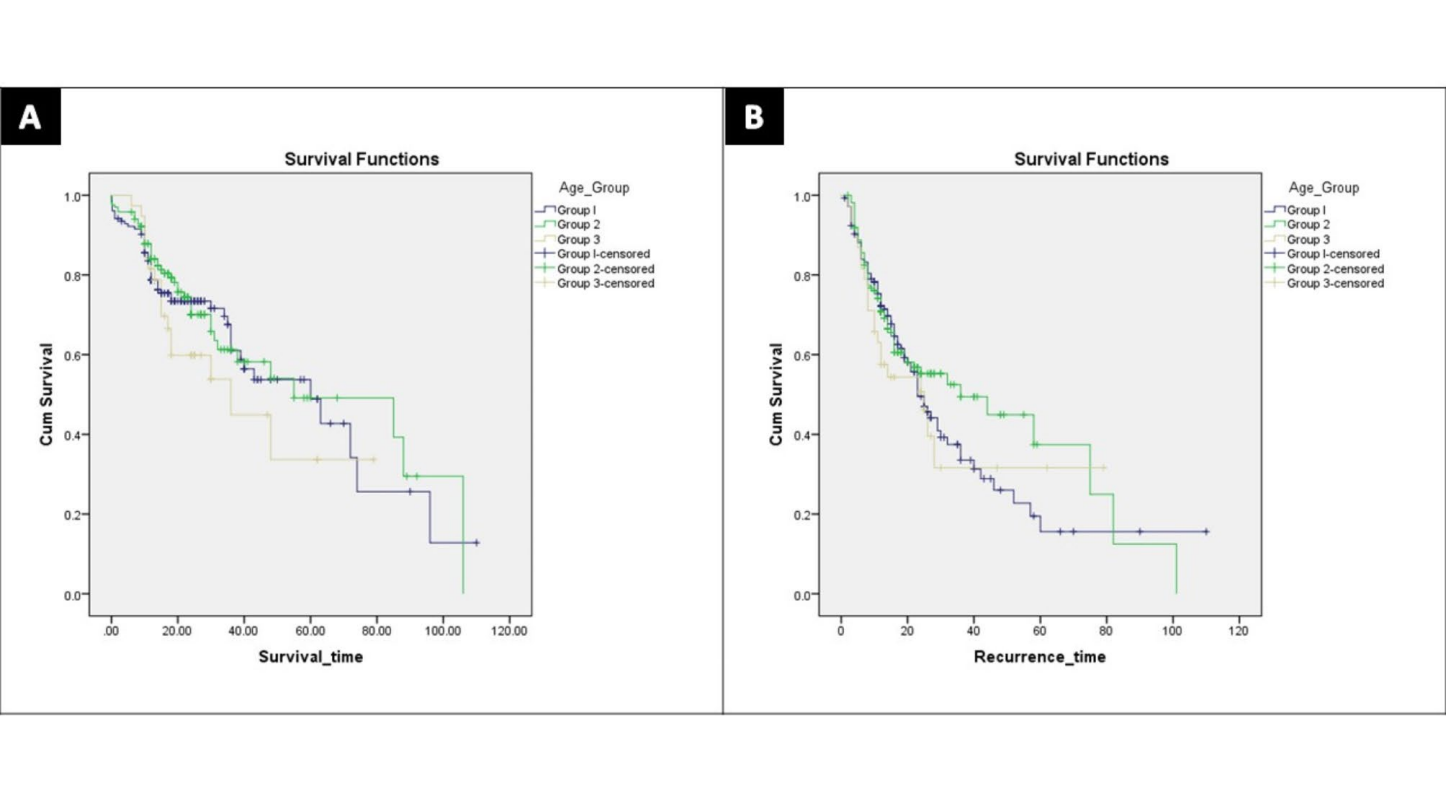
Survival curves of the study groups. **A**- Overall survival (Log Rank: Chi-Square = 1.981, df = 2, *p* = 0.371). **B**- Disease-free survival (Log Rank: Chi-Square = 1.538, df = 2, *p* = 0.464)

The 1-, 3-, and 5-years OS rates of Group I were 93.5%, 67.6%, and 48.4%, and 87.8%, 61.3%, and 49.2% for Group II, and 81.6%, 44.9%, and 33.7% for Group III, respectively (Log Rank: Chi-Square = 1.981, df = 2, *p* = 0.371) (Fig. 2-A).

#### Disease-free survival

Recurrence occurred in 165 patients (45.3%). Table provides a summary of the recurrence data (6). The 1-, 3-, and 5-years DFS rates of the whole patients were 73.4%, 42.8%, and 27.6.%, respectively (Fig. 1-B).

The 1-, 3-, and 5-years DFS rates of Group I were 75.3%, 37.5%, and 22.7%, and 74.1%, 49.4%, and 37.4%, for Group II, and 63%, 31.6%, and 31.6%, for Group III, respectively (Log Rank: Chi-Square = 1.538, df = 2, *p* = 0.464) (Fig. 2-B).

## Discussion

HCC is the most common primary malignancy affecting the liver [5, 6]. HCC is one of the most prevalent tumors in Egypt owing to the high prevalence of hepatitis C virus. Currently, the Egyptian Ministry of Health considered HCC as one of the most challenging health problems among the Egyptian people [27].

Liver transplantation and resection are curative therapy options for patients with HCC. The availability of living or cadaveric donors, high expenses, and the burden of immunosuppressive drugs are the drawbacks of liver transplantation. Thus, liver resection continues to be one of the most often utilized curative approaches for patients with HCC, with favorable perioperative and survival results [28, 29].

In the recent decades, a global increase in the life expectancy has been observed leading to a global aging phenomenon. This is mainly attributed to the improvement of general health conditions and social conditions, better work circumstances, and the development of governmental programs to support the elderly [30, 31]. In the context of cancer management, aging represents a major challenge. It is well known that old age is a well-established risk factor for the development of hepatobiliary malignancies including HCC. Globally, the number of older people with HCC has been rising. In Japan, more than 50% of patients undergoing liver resection for HCC are older than 70 years [3, 4]. Liver resection is still a crucial part of treating these elderly individuals because liver transplantation is not easily accessible to them due to a lack of donors and age-related comorbidities.

The definition of elderly patients has been variable among the different studies reflecting the differences in the health-status, social conditions, and the workforce-related functional ability [32]. Studies from Japan, a country with high prevalence of HCC, utilized the age of 75 years as a definition of elderly patients [33–35]. Other studies utilized the cut off of 70 years for definition of elderly patients [36, 37]. Other studies from the United Kingdom utilized the cut off of 80 years for definition of elderly patients undergoing resection for HCC owing to the improvement of the life expectancy in many Western countries [38]. Due to the fact that Egypt’s retirement age is linked to a considerable decline in both physical and mental health, the Egyptian government has adopted the chronological age of 60 as the definition of the aged [15, 16]. In the current study, the median age of the study patients was 61 years [18,80]. We evaluated our center experience of liver resection for HCC in elderly patients (exceeding 60 years) in comparison with younger patients. Moreover, to assess how the age of the patients affects the immediate and long-term results following liver resection for HCC. To the best of our knowledge, there is a paucity of information regarding the results of liver resection for hepatitis C virus-related HCC in older Egyptian patients.

Advanced patients’ age is usually associated with different physiological and functional changes that may limit the ability of older patients to withstand aggressive surgical therapies. Liver resection is a highly invasive complex therapy which is associated with several perioperative complications. Surgeons should balance between the potential risks of liver resection and the improved survival after curative liver resection. Proper patient selection is a crucial step for favorable outcomes of liver resection for HCC. The outcomes complicated liver resections had dramatically improved in the past years [22]. There were no appreciable variations observed in the preoperative demographics, radiological, or endoscopic data between the groups in the present research. The sole occasion that Group III’s preoperative serum creatinine was significantly higher, suggesting that aging has an effect on renal function. The groups did not significantly differ in terms of postoperative renal problems. Group III participants did not have any kidney problems following surgery.

In the setting of liver cirrhosis, liver resection is a technically challenging procedure which is associated with significant blood loss and transfusion requirements. It was assumed that elderly patients will receive more limited resections to preserve liver parenchyma while younger patients can tolerate more aggressive liver resections [38]. Pathologically confirmed liver cirrhosis was found in 345 patients (94.8%) in the current study. More major liver resections were performed in Group I compared to Groups II and III, however this was statistically non-significant. A significantly longer operation time was found in Groups II and III. Less perioperative transfusion requirements were found in Group III.

In the current study, shorter hospital stay was noticed in Group III patients denoting acceptable postoperative recovery and restoration of oral feeding. Wu et al. found a significantly longer hospital stay among elderly patients [39]. Sanyal et al. addressed similar hospital stay in both younger and older groups indicating that the application of enhanced recovery programs were equally applicable in both groups [38].

Previous studies had reported morbidity rate after liver resection for HCC in elderly patients ranging between 9 and 51% [13]. Several studies had shown comparable postoperative morbidity rate among elderly and younger patients in spite of the high incidence of age-related comorbidities among elderly patients [33, 40]. Other studies reported higher major postoperative complications among the elderly patients after liver resection for HCC especially respiratory, cardiac, renal and neurological complications [35, 41]. Interestingly, Ferrero et al. addressed lower postoperative complications among elderly patients undergoing liver resection for HCC [42]. In the current study, Group III patients experienced significantly less overall postoperative complication rate, especially post-hepatectomy liver dysfunction. Post-hepatectomy liver dysfunction is one of the commonest liver-specific morbidities after liver resection for HCC [43]. Our group previously addressed that the Egyptian HCC patient experienced higher incidence of liver dysfunction following liver resection for HCC [8]. In the current study, 144 patients (39.6%) experienced post-hepatectomy liver dysfunction, however the main proportions were grade A dysfunction (83 patients – 22.8%). Grade III patients experienced lower incidence of post-hepatectomy liver dysfunction and all of them were grade A dysfunction (7 patients – 7%). This may be attributed to more precise patient selection and more limited liver resections to preserve the liver parenchyma applied to Group III patients. Hence, less early postoperative mortality was noted in Group III from post-hepatectomy liver failure.

The primary factor influencing a patient’s prognosis after liver resection is postoperative HCC recurrence. Recurrence of HCC is linked to detrimental effects on surgeons as well as patients. Similar recurrence and DFS rates were found in earlier research on liver resection for HCC in elderly individuals [33, 35, 40, 41]. There were 165 patients (45.3%) who experienced an HCC recurrence in the current study, and there were no differences in the DFS rates across the groups.

Long-term survival outcomes are the most essential outcome in cancer management. Several studies had reported 5-years OS rates after liver resection for HCC in elderly patients ranging between 26 and 57.9%. Similarly, previous studies about liver resection for HCC in elderly patients reported comparable OS rates between younger and older patients [33, 40, 42, 44, 45, 46].

The present research has certain shortcomings. Due to the retrospective nature of the study, which only looked at one location, patient selection bias may exist. In comparison to other studies conducted in developed nations, the number of patients above the age of 70 is quite small. A larger patient sample size and multicenter study in the future should address these shortcomings.

## Conclusion

In conclusion, certain elderly individuals with HCC may safely undergo curative liver resection. The perioperative, oncological, and survival outcomes after liver resection for older patients with HCC in this study were comparable to those of younger patients. For older patients with HCC, careful preoperative evaluation, surgical methods, and postoperative care are essential for accomplishing favorable results. Curative liver surgery should not be prohibited based only on an advanced patient’s age.

## Data Availability

No datasets were generated or analysed during the current study.
